# Factors affecting the concentration of metals and metalloids in the kidneys of a top predator, the Eurasian Buzzard (*Buteo buteo*) wintering in farmland in Poland

**DOI:** 10.1007/s11356-024-35378-0

**Published:** 2024-10-29

**Authors:** Ignacy Kitowski, Dariusz Jakubas, Dariusz Wiącek, Grzegorz Pitucha, Agnieszka Sujak

**Affiliations:** 1 University College of Applied Sciences in Chełm, Pocztowa 54, 22-100 Chełm, Poland; 2 Department of Vertebrate Ecology and Zoology, Faculty of Biology, University of Gdańsk, Wita Stwosza 59, 80-308 Gdańsk, Poland; 3 Bohdan Dobrzański Institute of Agrophysics of the Polish Academy of Sciences, Doświadczalna 4, 20-290 Lublin, Poland; 4 Biodiversity Laboratory, Institute of Agricultural Sciences, Land Management and Environmental Protection, University of Rzeszów, Ćwiklińskiej 1A, 35-601 Rzeszów, Poland; 5 Department of Biosystem Engineering, Faculty of Environmental Engineering and Mechanical Engineering, Poznań University of Life Sciences, Wojska Polskiego 50, 60-627, Poznań, Poland

**Keywords:** Bioaccumulation, Metals, Metalloids, Kidney, Eurasian Buzzard (*Buteo buteo*)

## Abstract

During late autumn and winter, raptors in the western Palearctic face challenges due to food scarcity and dropping temperatures. That time they can be exposed to various elements including toxic ones ingested with food. Kidney samples from 22 females and 19 males of a medium-sized raptor, the Common Buzzard *Buteo buteo* found dead in farmland of Eastern Poland in winter were analyzed for a concentration of 21 elements. Elemental concentrations were analyzed regarding the age and sex of birds. Results revealed that only 4.9% of individuals had kidney lead levels exceeding 8.0 mg, while 9.8% showed cadmium levels above 8.0 mg/kg, indicating potential poisoning. The study also highlighted the limited entry of arsenic into agricultural ecosystems exploited by Common Buzzards. Sex differences were noted, with females accumulating more lead and vanadium than males which can be associated with foraging niche partitioning between sexes driven by body size dimorphism. Sulfur showed complex interactions with cadmium, mercury, and zinc, with a positive correlation between sulfur and zinc levels in the kidneys, emphasizing dietary needs during food scarcity. A positive correlation was found between zinc and lead concentrations, indicating zinc’s role in mitigating lead’s impact. The study also revealed positive correlations between selenium and highly toxic elements like mercury (Spearman correlation, *r*_s_ = 0.41) and cadmium (*r*_s_ = 0.51), suggesting a mitigating effect of selenium on exposure to heavy metals. This study enhances understanding of year-round environmental contamination exposure for raptors and sheds light on bioaccumulation in a top predator.

## Introduction

Raptors are top predators in food webs and therefore are capable of accumulating both toxic and essential metals and metalloids (Carneiro et al. 2016; Krone 2018; Smits and Naidoo 2018; Badry et al. 2020; Monclus et al. 2020). The late autumn and winter periods are extremely challenging for raptors remaining in the Western Palearctic due to reduced food availability and dropping temperatures. Therefore, studying the accumulation of metals and metalloids in raptors’ key organs during this period may help understand their year-round exposure to environmental contaminants that could affect individual conditions and survival.

Some toxic elements may biomagnify in organisms of top predators. Such elevated concentrations may have a negative influence on their health status, reproductive performance, survival, and in turn, on population dynamics (Carneiro et al. 2016; Smits and Naidoo 2018). Some other life history traits and features like long lifespan, often manifested sexual dimorphism, territoriality during the breeding season, exploitation of different types of prey (Negro and Galván 2018; Tapia and Zuberogoitia 2018; Monclus et al. 2020) may also be the reason for variation in both toxic and essential metals and metalloids concentration (Smits and Naidoo 2018; Badry et al. 2020; Rodríguez-Álvarez et al. 2022).

Diet of some birds of prey is opportunistic, and thus, they may hunt for prey in various habitats, exposed to varying environmental and organism contamination levels. The Common Buzzard (*Buteo buteo*) is an example of such an opportunistic bird of prey. Its diet is variable, with small mammals serving as the main component (Jedrzejewski et al. 1994; Goszczynski 1997; Goszczynski et al. 2005; Wuczyński 2003, 2005). This species exploits agricultural landscapes in many European countries during the non-breeding season, rarely venturing into suburban areas and ecotones between forests and fields. Unfortunately, agrarian habitats are often contaminated by agrochemicals (Alengebawy et al. 2021; Toth et al. 2016). Some authors have indicated that the Common Buzzard can be considered a suitable model for continental-scale biomonitoring due to its widespread distribution and population abundance (Badry et al. 2020).

The kidneys, unlike the livers, are less frequently used for bioindication purposes, although this organ performs many vital functions in vertebrate organisms (Kalisinska et al. 2014). Kidney function is associated with homeostasis, excretion of harmful metabolites in urine, regulation of body fluid volume, and blood pH. Additionally, the kidneys influence the production processes of certain vitamins (Braun 1993; Finco 1997; Braun 2015; Reece 2015; Braun and Lefebvre 2008; Reece 2015). Although the kidneys do not play as significant a role as the liver in the detoxification of some elements (Ikemoto et al. 2004; Barbier et al. 2005; Scheuhammer et al. 2007; Dias dos Santos et al. 2021; Jakimska et al. 2011), they are attributed with a certain ability to accumulate toxic metals, especially cadmium (Garcia-Fernandez et al. 1995; Tomza-Marciniak et al. 2019; Vizuete et al. 2019). From a bioindication perspective, it has been also emphasized that the determination of concentrations in both the liver and kidney, rather than in the liver alone, may increase the likelihood of identifying cases of poisoning or high exposure to certain elements, for example, lead (Wayland et al. 1999).

In this study, we investigated factors affecting metals and metalloid concentration in the kidneys of Common Buzzards found dead in late autumn and winter in Eastern Poland. We expected differences between age (adults, immature) and sex category groups. Given age differences in hunting efficiency in raptors (Toland 1986; Ellis et al. 1993; Schindler 2002; Rutz et al. 2005), we expect that less experienced immatures may hunt more frequently in suboptimal areas and/or forage on suboptimal prey (e.g., carrion) compared to more experienced adults. It may make immatures more exposed to lead contamination. Given a reverse sexual dimorphism in Common Buzzards (Cramp and Simmons 1980; Manosa and Cordero 1992; Walls and Kenward 2020), we also expect some sex differences in exposure to contamination as a consequence of possible diet differences driven by inter-sex foraging niche partitioning. On the other hand, a previous study of trace element concentration in Common Buzzards revealed age but not sex differences in livers (Kitowski et al. 2017b). Given various agricultural techniques used in larger farms with cultivation monocultures compared to smaller patches of arable lands, we expect higher concentrations of agrochemical-derived heavy metals in organisms living in intensively cultivated larger farms. In turn, we may expect higher contamination of Common Buzzards preying on species living in larger monocultures compared to ones living in the mosaics of smaller arable lands.

## Materials and methods

### Samples collection

**Fig. 1:**
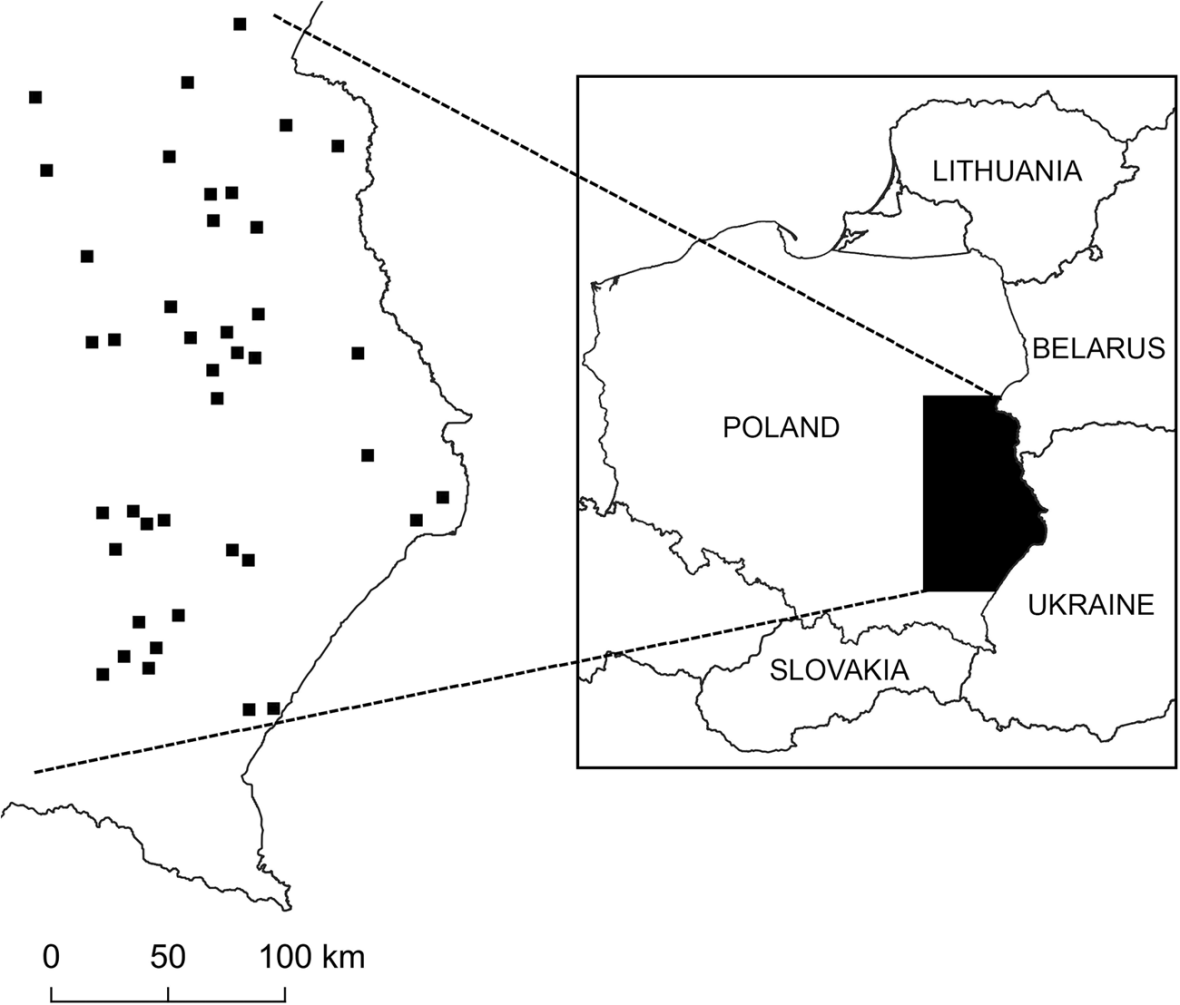
Study area with location of all sampling sites

In total, 41 kidneys were sampled from dead Common Buzzards collected in an agricultural landscape in Eastern Poland (Lublin, Rzeszow, and Warsaw and Białystok regions; Fig. 1). The whole studied area is characterized by a predominance of the rural landscape with the scarce presence of industrialized zones.

The kidneys were collected from deceased birds brought to veterinary clinics in winter—between December and March from 2010 to 2016. The birds were either freshly deceased (sometimes during transport to the veterinarian) or, if deemed untreatable upon arrival, were euthanized by veterinary doctors to prevent unnecessary suffering. None of the birds remained in the clinics for more than five days. The cause of death was often difficult to determine, but over 70% of the cases were clearly due to poisoning by carbofuran and other carbamate insecticides, collisions with road and railway vehicles, or collisions with high-voltage power lines. None of the birds in the study were intentionally shot. After being extracted from the birds, the kidneys were stored in freezers until analysis.

After dissection, birds were identified by internal examination and classified as either immature (2 years old or younger) or adult (older than 2 years) according to their plumage, gonadal development, and iris color (Cramp and Simmons 1980; Baker 1993; Forsman 1999; Walls and Kenward 2020).

### Laboratory analyses

The kidneys were extracted intact from the collected individuals and dissected within 1–2 h after death. Collected organs were stored in freezers until analyses. The mineral composition of kidneys was determined using Inductively Coupled Plasma Optical Emission Spectrometry (ICP-OES, iCAP Series 6500 Duo, Thermo Scientific, USA).

Before analyzing the metals and metalloids, the samples (0.5 g each) underwent mineralization using a Microwave Digestion System (Berghof Speedwave, Eningen, Germany). During the acid digestion process, each sample was monitored for optical changes, temperature, and pressure within Teflon vials (type DAP 100). For the digestion, 8 ml of 65% nitric acid (HNO_3_) was used to break down the kidney samples.

The mineralization process was carried out according to a specific protocol: an initial 10 min during which the temperature was gradually increased from room temperature to 140 °C, followed by 10 min at 140 °C. Then, the temperature was increased from 145 to 195 °C over 15 min, maintained at 195 °C for 10 min, and finally allowed to cool back to room temperature. Throughout the mineralization process, the pressure was kept below 20 bars. Once mineralization was complete, the clear solution was cooled to room temperature, transferred to 50-ml graduated flasks, and diluted with deionized water (ELGA Pure Lab Classic) up to the indicated level.

The ICP OES equipment was operated under the following conditions: an RF generator power of 1150 W, an RF generator frequency of 27.12 MHz, a coolant gas flow rate of 16 L/min, a carrier gas flow rate of 0.65 L/min, and an auxiliary gas flow rate of 0.4 L/min. The maximum integration time was set to 15 s, with a pump rate of 50 rpm, axial viewing configuration, three replicates per sample, and a flush time of 20 s. Calibration of element concentrations was performed using multi-element standards CCS-4, CCS-5, and CCS-6 (100 µg/ml in 7% HNO_3_, Inorganic Ventures, USA). Additional details regarding the laboratory analyses can be found in Kitowski et al. (2017a) and Klich et al. (2021).

### Statistical analyses

To characterize the main farmland type in the area where the birds were found, data from the Survey of Agricultural Population from 2020 available in the Central Statistical Office of Poland (Powszechny Spis Rolny 2021) was used. As a proxy of dominating farmland type in the vicinity of locations where Buzzards were collected, we used a proportion of farmland patches with an area above 15 ha dividing the farmland into two categories:

- monoculture—prevalence (> 50%) of farms with large patches of arable land (patch area > 15 ha), we expected higher more intense agriculture with intense use of agrochemicals
- mosaic—prevalence of farms with smaller patches of arable land (patch area ≤ 15 ha)

To validate the locations where the Buzzards were collected, data from particular administration units used in Powszechny Spis Rolny (2021) were taken.

To compare the concentrations of particular elements among sexes and ages and farmland type, two methods were used:

1. multivariate analyses for all elements simultaneously:
  - two-way PERMANOVA (non-parametric MANOVA based on the Bray–Curtis measure; Anderson 2001) with fixed factors (age and sex or habitat type and sex) and their interaction as explanatory variables; we were not able to implement all predictors at once so we first performed the analysis with age and sex, and after that (after finding the significant predictor) with sex and habitat type.
  - The similarity percentage breakdown (SIMPER) procedure to assess the average percentage contribution of individual factors to the dissimilarity between objects in a Bray–Curtis dissimilarity matrix (Clarke 1993).
2. univariate analysis for particular elements—a non-parametric Wilcoxon test (U Mann–Whitney test) was used.

To investigate the relationship between the concentration of the studied elements in the kidneys the Spearman correlation coefficient was used.

Multivariate analyses were performed on log-transformed data for all elements. All statistical analyses were performed in R software (R Core Team 2021) and PAST 4.11 (Hammer et al. 2001).

## Results

The following pattern of trace element concentration was found in the kidneys of all 41 collected Common Buzzard: S > K > Na > Fe > Ca > Mg > Zn > Cu > Mn > Se > Cd > Pb > Mo > Hg > V > Cr > Sr > Ni > As > Ba > Co (Table 1).

**Table 1:** Age dependencies of concentrations [mg/kg DW] of the elements in Common Buzzard females and males

	F_IMM (*n* = 6)		F_AD (*n* = 16)		M_IMM (*n* = 8)		M_AD (*n* = 11)		Total (* n* = 41)	
	Mean ± SD	Min–max	Mean ± SD	Min–max	Mean ± SD	Min–max	Mean ± SD	Min–max	Mean ± SD	Min–max
As	0.556 ± 1.013	0.049–2.616	0.175 ± 0.146	0.014–0.534	0.167 ± 0.089	0.038–0.299	0.222 ± 0.152	0.018–0.576	0.241 ± 0.402	0.014–2.616
Ba	0.083 ± 0.045	0.021–0.158	0.144 ± 0.114	0.016–0.457	0.103 ± 0.055	0.008–0.183	0.190 ± 0.116	0.049–0.430	0.139 ± 0.103	0.008–0.457
Ca	834.8 ± 772.2	248.2–2301	664.4 ± 456.6	240.2–1784	385.2 ± 124.9	193.4–539.8	631.9 ± 319.8	308.5–1434	626.1 ± 446.9	193.4–2301
Cd	1.014 ± 0.706	0.435–2.315	2.751 ± 2.549	0.218–10.32	1.494 ± 1.107	0.441–3.024	5.042 ± 5.417	0.424–19.17	2.866 ± 3.500	0.218–19.17
Co	0.042 ± 0.034	0.008–0.099	0.071 ± 0.099	0.012–0.416	0.051 ± 0.026	0.009–0.086	0.071 ± 0.013	0.051–0.090	0.062 ± 0.064	0.008–0.416
Cr	1.250 ± 0.437	0.678–1.626	1.161 ± 0.368	0.755–1.904	1.143 ± 0.297	0.749–1.462	1.227 ± 0.219	0.791–1.518	1.187 ± 0.322	0.678–1.904
Cu	12.42 ± 3.171	8.613–17.68	11.08 ± 3.283	5.824–16.10	13.67 ± 7.782	7.614–27.28	12.53 ± 8.948	6.622–38.63	12.17 ± 6.074	5.824–38.63
Fe	961.5 ± 432.8	511.7–1685	1127 ± 836.5	226.9–2626	1117 ± 746.4	445.6–2502	741.9 ± 365.8	381.8–1564	997.4 ± 666.7	226.9–2626
Hg	1.529 ± 0.837	0.802–2.920	1.731 ± 1.031	0.041–3.918	1.787 ± 1.400	0.476–4.815	2.760 ± 3.212	0.245–10.18	1.988 ± 1.908	0.041–10.18
K	10524 ± 1361	8959–12410	11154 ± 1954	8385–15760	9665 ± 927.8	8528–11180	10557 ± 1600	8815–14400	10611 ± 1658	8385–15760
Mg	674.9 ± 259.7	390.2–1127	581.4 ± 180.4	363.1–955.1	568.2 ± 124.6	374.3–775.5	624.8 ± 99.99	427.0–798.3	604.1 ± 164.8	363.1–1127
Mn	13.06 ± 7.364	6.253–26.12	8.605 ± 5.302	2.449–20.49	7.133 ± 3.543	3.682–12.37	7.136 ± 4.793	2.146–17.66	8.575 ± 5.409	2.146–2612
Mo	1.760 ± 0.593	1.302–2.937	2.254 ± 0.975	1.068–4.692	2.120 ± 0.468	1.505–2.996	2.422 ± 0.506	1.713–3.395	2.200 ± 0.740	1.068–4.692
Na	48756 ± 2156	2089–7942	4441 ± 1718	2767–9189	5434 ± 2591	2381–9030	4957 ± ± 1556	3173–8556	4836 ± 1898	2089–9189
Ni	0.722 ± 0.184	0.503–0.975	1.006 ± 1.408	0.499–6.272	0.603 ± 0.128	0.455–0.806	0.619 ± 0.070	0.504–0.715	0.782 ± 0.887	0.455–6.272
Pb	2.403 ± 0.495	1.852–3.155	4.338 ± 7.670	0.900–32.33	0.893 ± 0.385	0.414–1.552	1.214 ± 0.480	0.427–1.716	2.544 ± 4.950	0.414–32.33
S	12943 ± 2132	10660–16230	11408 ± 2728	8469–20100	9539 ± 692.5	8179–10,310	10387 ± 1245	8770–13320	10993 ± 2234	8179–20100
Se	2.807 ± 1.254	1.286–4.560	3.602 ± 1.025	2.191–6.053	3.667 ± 1.106	1.934–5.408	3.726 ± 0.623	2.543–4.663	3.531 ± 0.998	1.286–6.053
Sr	1.161 ± 0.379	0.662–1.629	1.015 ± 0.485	0.500–2.249	0.769 ± 0.252	0.466–1.522	0.670 ± 0.365	0.347–1.627	0.895 ± 0.441	0.347–2.249
V	2.629 ± 0.557	2.000–3.368	1.979 ± 0.730	0.489–2.706	1.240 ± 0.984	0.195–2.523	0.949 ± 0.835	0.044–2.714	1.653 ± 0.969	0.044–3.368
Zn	108.8 ± 25.86	78.88–150.5	103.7 ± 57.96	41.15–288.8	81.36 ± 28.32	52.91–143.8	89.73 ± 34.04	58.46–175.0	96.34 ± 43.30	41.15–288.8

*F_IMM*, immature female; *M_IMM*, immature male; *F_AD, *adult female; *M_AD*, adult male

When age, sex, and their interaction were considered, the concentrations of all studied elements in kidneys differed significantly between sexes (PERMANOVA based on Euclidean similarity: *F*_1,37_ = 3.607, *p* = 0.0008). No significant influence of age (*F*_1,37_ = 1.757, *p* = 0.079) or age × sex interaction on element concentrations (*F*_1,37_ = 0.718, *p* = 0.687) was found.

When farmland category, sex, and their interaction were considered, the concentrations of all studied elements in kidneys differed significantly between sexes (PERMANOVA based on Euclidean similarity: *F*_1,37_ = 3.619, *p* = 0.001). No significant influence of farmland category (*F*_1,37_ = 1. 501, *p* = 0.145) or farmland category × sex interaction on element concentrations (*F*_1,37_ = 1.106, *p* = 0.355) was found.

SIMPER analysis revealed that V, Cd, As, Hg, Pb, Ba, and Co contributed the most (13.9%, 12.0%, 11.5%, 10.6%, 9.5%, 8.4%, and 7.4%, respectively) to the pattern of overall inter-sex dissimilarity observed in elemental concentrations.

**Fig. 2:**
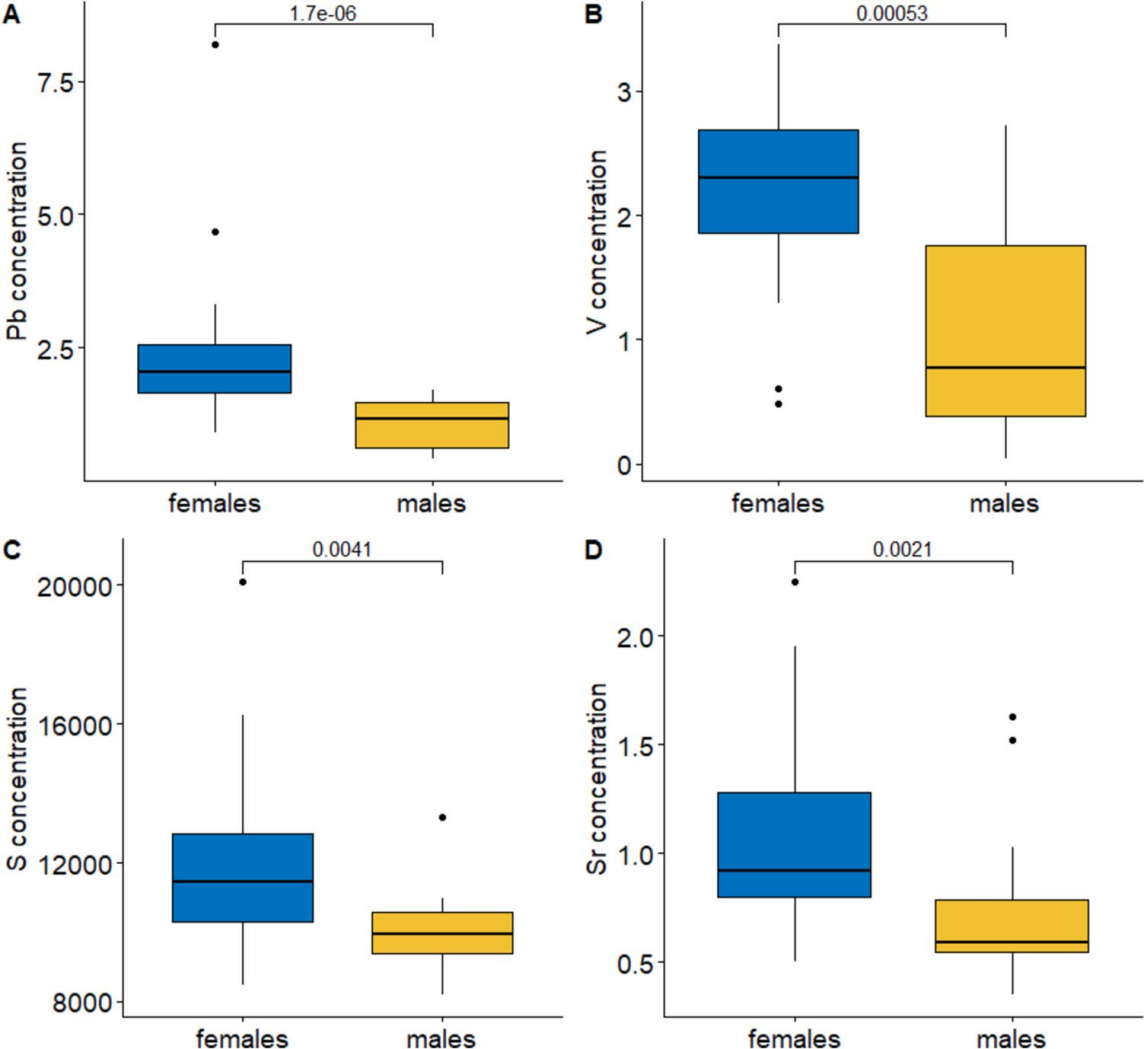
Sex differences in Pb (**A**), V (**B**), S (**C**), and Sr (**D**) concentration in kidneys of Common Buzzards spending late autumn and winter in **E** Poland. Boxplots show the median (band inside the box), the first (25%) and third (75%) quartile (box), the lowest and the highest values within the 1.5 interquartile range (whiskers), and outliers (dots). The values above lines indicate p values for the Wilcoxon test

Univariate analyses performed separately for particular elements revealed significant sex effects for four elements (Wilcoxon tests, *p* < 0.003) with females having significantly higher Pb, V, S, and Sr concentrations than males (Fig. 2 and Table 1). Females also tended to have higher levels of Ni and Mn and lower levels of Co compared to males (0.06 > *p* < 0.09) (Fig. 2 and Table 1). Concentrations of other studied elements did not differ significantly between the sexes (*p* > 0.13).

**Fig. 3:**
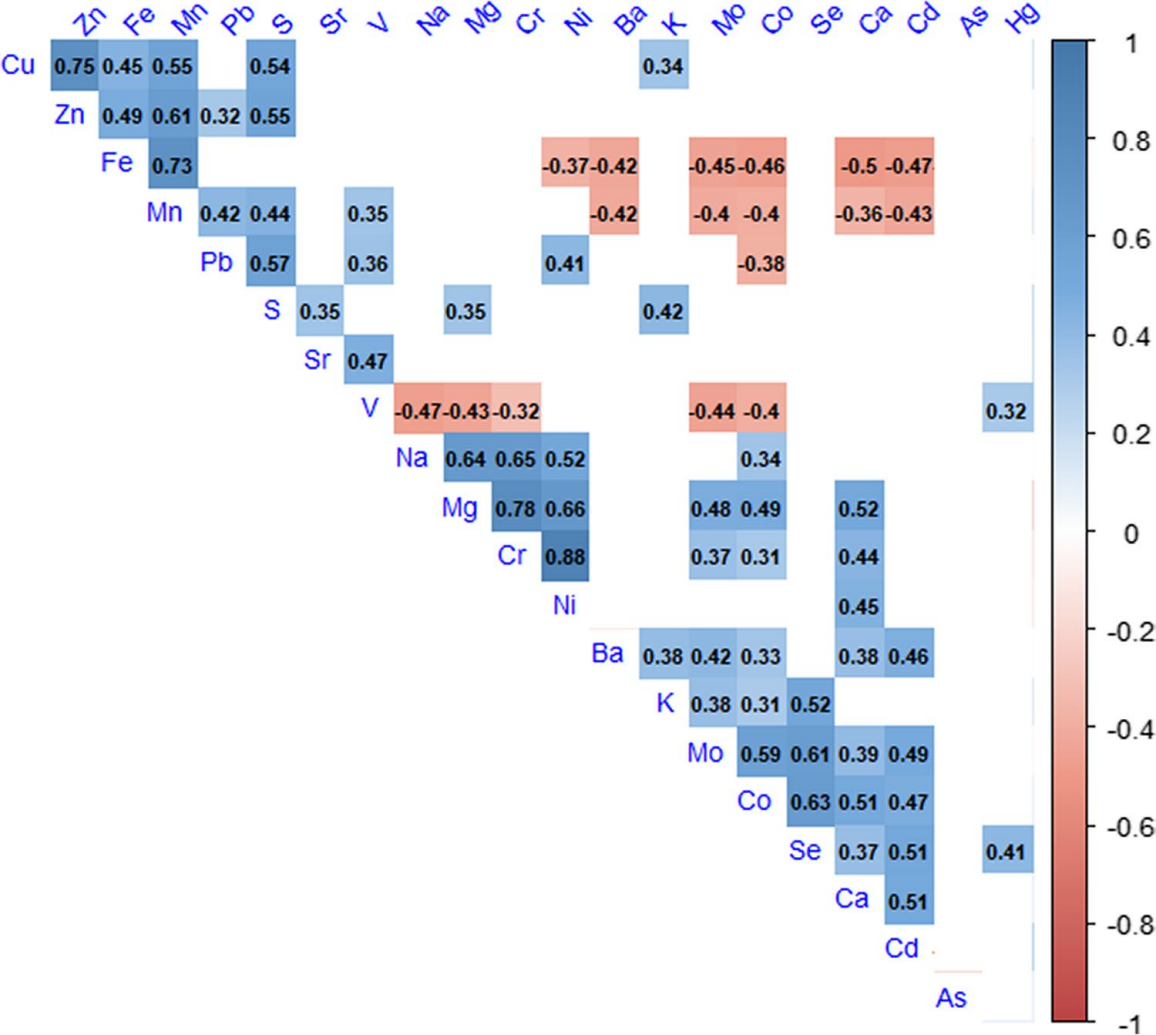
Spearman correlation coefficients (*r*_s_) for all studied elements in the kidneys of Common Buzzards spending late autumn and winter in E Poland. The color indicates the strength of the correlation. Only significant correlations (*p* > 0.05) are shown

Some significant relationships between elements concentrations in kidneys were found (Spearman rank correlation) (Fig. 3). The strongest correlations (*r*_s_ ≥ 0.75) were found for Cu and Zn (0.75), Mg and Cr (0.78), and Cr and Ni (0.88). All negative correlations were weaker (*r*_s_ < 0.5) (Fig. 3).

## Discussion

To our knowledge, this is the first study on kidney concentrations of metals and metalloids in Common Buzzards from Central Europe and one of the few studies to investigate the concentrations of metals and metalloids in this species concerning sex and age (Naccari et al. 2009; Licata et al. 2010; Carneiro et al. 2014). This paper presents elemental concentrations in the kidneys of a raptor species that spends its challenging late autumn and winter periods in the agricultural landscape of eastern Poland.

In contrast to the liver, avian kidneys are not involved in key metabolic processes associated with the uptake and storage of trace elements (Boden et al. 2007; Zaefarian et al. 2019). The primary role of the kidneys is the excretion of nitrogenous waste products and they play a central role in body water and solute homeostasis (Braun 1998). Thus, in contrast to other ecotoxicological studies on raptors’ livers (Zaccaroni et al. 2003; Kim and Oh 2016; Kitowski et al. 2017b), we only found intergroup differences in kidney concentrations for two elements (see details below). We also did not find a significant relationship between the concentrations of metals and metalloids and variables describing the Common Buzzard foraging habitats. Below, we discuss factors influencing the concentrations of particular elements.

## Lead

Unlike the bones, which reflect long-term exposure to lead quite well (Pain et al. 2005; Komosa and Kitowski 2008; Ishii et al. 2018), the kidneys and livers may serve as good indicators of current, short-term exposure to this element (Krone 2018; Monclus et al. 2020; Kitowski et al. 2024).

There are a few proposed thresholds of toxication with Pb for avian kidneys. According to Wayland et al. (1999), kidney concentration of 5.0 mg/kg DW represents elevated exposure in raptors. Jager et al. (1996) have reported that 10 mg/kg DW lead in the kidneys of Common Buzzard corresponds to environmental pollution. Wayland et al. (1999) have estimated that a kidney concentration of 18 mg/kg DW corresponded to a liver concentration of 30 mg/kg DW, reflecting severe clinical lead poisoning. In further studies, Wayland et al. (2003) considered Golden Eagles *Aquilla chrysaetos* and Bald Eagles *Haliaeetus leucocephalus* with kidney lead concentration > 20 mg/kg DW as poisoned, and individuals with levels > 6 mg/kg DW as individuals with elevated lead concentrations.

Only two individuals among the studied Common Buzzards (4.9%) had elevated kidney lead levels (8.18 mg/kg DW and 32.33 mg/kg DW), indicating that both birds exceeded the threshold suggestive of at least subclinical toxicity. Following the thresholds proposed by Kim and Oh (2012), we attribute the death of the second individual to high concentrations of lead in the kidneys especially since its gizzard contained 4 lead pellets.

In contrast to previous studies on raptors including Common Buzzards showing significant effects of age and/or age-sex interaction on lead concentration in kidneys and livers (Zaccaroni et al. 2003; Kim and Oh 2016; Kitowski et al. 2017b), we did not find such significant effects.

Other studies on buzzards have also found no indication of significant differences in accumulation levels in the kidney or the liver (Pb liver, muscles), between adult and juvenile Common Buzzards (Jager et al. 1996; Naccari et al. 2009; Licata et al. 2010; Castro et al. 2011). Solely, Carneiro et al. (2014) in Common Buzzards from Portugal found differences in blood Pb concentrations between adults and juveniles.

We only found that females accumulated more lead in kidneys than males (Fig. 2, Table 2). This effect might be explained by reverse sexual dimorphism (RSD) expressed in larger size of females compared to males **(**Cramp and Simmons 1980; Walls and Kenward 2020), commonly present in raptors (Schoenjahn et al. 2020; Wang et al. 2023). It may lead to at least partial inter-sex foraging niche partitioning. Larger females may be able to outcompete smaller males and other species of scavengers like corvids thus being more prone to lead intake by foraging on carrion of animals shot with lead ammunition.

**Table 2:** Concentrations [mg/kg DW] of the elements in Common Buzzards. Data for age and sex of all the examined individuals

	AD (*n* = 27)		IMM (*n* = 14)		M (*n* = 19)		F (*n* = 22)	
	Mean ± SD	Min–max	Mean ± SD	Min–max	Mean ± SD	Min–max	Mean ± SD	Min–max
As	0.194 ± 0.148	0.014–0.576	0. 334 ± 0.662	0.0382–2.616	0.199 ± 0.129	0.018 –0.576	0.279 ± 0.538	0.014–2.616
Ba	0.163 ± 0.115	0.016–0.457	0.094 ± 0.050	0.008–0.183	0.154 ± 0.103	0.008–0.430	0.127 ± 0.103	0.016–0.457
Ca	651.1 ± 399.8	240.2–1784	577.9 ± 539.5	193.4–2301	528.0 ± 280.2	193.4–1434	710.7 ± 544.9	240.2–2301
Cd	3.684 ± 4.043	0.218–19.170	1.288 ± 0.955	0.435–3.024	3.548 ± 4.474	0.424–19.17	2.277 ± 2.321	0.218–10.32
Co	0.071 ± 0.075	0.012–0.416	0.047 ± 0.029	0.008–0.099	0.062 ± 0.022	0.009–0.090	0.063 ± 0.086	0.008–0.416
Cr	1.188 ± 0.313	0.755–1.904	1.189 ± 0.352	0.678–1.626	1.191 ± 0.250	0.749–1.518	1.185 ± 0.380	0.678–1.904
Cu	11.669 ± 6.128	5.82–38.63	13.133 ± 6.10	7.614–27.28	13.01 ± 8.27	6.622–38.63	11.44 ± 3.23	5.824–17.68
Fe	700.0 ± 701.7	226.9–2626	1050.4 ± 615.2	445.6–2502	899.0 ± 572.0	381.8–2502	1081.7 ± 741.7	226.9–2626
Hg	2.150 ± 2.202	0.041–10.18	1.68 ± 1.16	0.475–4.82	2.350 ± 2.596	0.245–10.18	1.676 ± 0.967	0.041–3.918
K	10911 ± 1810	8385–15760	10033 ± 1171	8528–12410	10182 ± 1401	8528–14400	10982 ± 1803	8385–15760
Mg	599.1 ± 151.9	363.1–955.1	613.9 ± 193.1	374.3–1127	601.0 ± 111.5	374.3–798.3	606.9 ± 202.7	363.1–1127
Mn	8.007 ± 5.059	2.146–20.490	9.672 ± 6.072	3.682–26.12	7.135 ± 4.200	2.146–17.66	9.820 ± 6.092	2.449–26.12
Mo	2.322 ± 0.809	1.068–4.692	1.966 ± 0.536	1.302–2.996	2.295 ± 0.501	1.505–3.395	2.119 ± 0.902	1.068–4.692
Na	4651 ± 1643	2767–9189	5194.5 ± 2342	2089–9030	5158 ± 2003	2381–9030	4559 ± 1804	2089–9189
Ni	0.849 ± 1.088	0.499–6.272	0.654 ± 0.160	0.455–0.979	0.612 ± 0.096	0.455–0.806	0.929 ± 1.200	0.499–6.272
Pb	3.065 ± 6.040	0.427–32.33	1.540 ± 0.880	0.414–3.155	1.079 ± 0.461	0.414–1.716	3.810 ± 6.547	0.900–32.33
S	10992 ± 2270	8469–20100	10998 ± 2250.2	8179–16230	10030 ± 1110	8179–13320	11827 ± 2625	8469–20100
Se	3.653 ± 0.871	2.191–6.053	3.298 ± 1.208	1.286–5.408	3.701 ± 0.832	1.934–5.408	3.386 ± 1.121	1.286–6.053
Sr	0.874 ± 0.466	0.347–2.249	0.937 ± 0.403	0.466–1.629	0.712 ± 0.353	0.347–1.627	1.055 ± 0.455	0.500–2.249
V	1.560 ± 0.917	0.044–2.714	1.835 ± 1.072	0.195–3.368	1.072 ± 0.886	0.044–2.714	2.157 ± 0.737	0.489–3.368
Zn	98.0 ± 49.32	41.15–288.8	93.11 ± 29.78	52.91–150.5	86.21 ± 31.203	52.91–175.0	105.1 ± 50.63	41.15–288.8

*AD*, adults; *IMM*, immatures; *M*, males; *F*, females

In this study, Common Buzzards that died during the autumn–winter period accumulated 1.56 mg/kg DW of lead in their kidneys. Other authors have reported kidney lead concentrations in the same species ranging from 0.39 to 1.9 mg/kg DW (Hontelez et al. 1992; Jager et al. 1996; Licata et al. 2010; Castro et al. 2011; Carneiro et al. 2014).

Recently (in 2023), a ban on the use of Pb gunshot in or around wetlands has been introduced in the European Union (Commission Regulation EU 2021). This measure is expected to protect species of raptors strongly associated with wetlands, such as the White-tailed Eagle or Marsh Harrier (*Circus aeruginosus*) and Anatidae from lead poisoning. However, it will not protect Common Buzzards and other raptors or scavengers (such as corvids) foraging in agricultural landscapes from lead ingestion when feeding on wounded or dead animals, primarily in non-wetland habitats. The Common Buzzard is the most widespread raptor species in agricultural landscapes during winter in East Poland (Krason and Michalczuk 2019; Polak 2021). Implementing a total ban on lead ammunition use in all habitats (as in other European countries) is the only effective measure to protect raptors and scavengers from lead poisoning originating from this source (Sonne et al. 2022; Kitowski et al. 2024).

## Cadmium

Cadmium is considered a dangerous nephrotoxic element. When transported, cadmium complexes with methylthionine are easily filtered in the kidney glomeruli and reabsorbed in the proximal tubules. However, upon its decay, Cd^2+^ ions begin to act toxically, even in small amounts, leading to kidney disorders, including mild tubular atrophy (Klaassen et al. 1999; Świergosz and Kowalska 2000; Genchi et al. 2020; Barregard et al. 2022). Kidneys have been recognized as the primary organ for cadmium accumulation in birds (61%), followed by the liver (31%), brain (4%), bones (3.5%), and blood (0.5%) (Garcia-Fernandez et al. 1995). On the other hand, some other studies have suggested a higher contribution of the liver to this element accumulation (approximately 50% of the total Cd burden) (Vizuete et al. 2019).

Cadmium concentration > 8.0 mg/kg DW in avian kidneys is consistent with poisoning in wild birds (Scheuhammer 1987). This means that 4 (9.8%) of the buzzards examined in this study could have been poisoned by this element. Study on owl, the Brown Hawk Owl (*Ninox scutulata*) has found that 2 (27.0%) out of 7 examined individuals accumulated > 8 mg/kg DW cadmium in the kidneys (Kim and Oh 2012).

Age and sex have been found as factors explaining cadmium accumulation in avian kidneys (Saeki et al. 2000; McFarland et al. 2002; Kalisińska et al. 2004; Lucia et al. 2010; Berglund et al. 2011; Barrales et al. 2021; Vizuete et al. 2022). However, we did not observe such intergroup differences in the examined Common Buzzards, as in some other studies on various avian species (Stewart et al. 1999; Nam et al. 2005; Hoshyari et al. 2015). However, the studies on Common Buzzards from Italy (Naccari et al. 2009) and Portugal (Carneiro et al. 2014) have demonstrated significant age differences with higher cadmium levels in adults compared to immatures.

Previous studies seem to indicate the existence of geographic patterns regarding the accumulation of cadmium in the kidneys of Common Buzzards. Birds from the northern part of Europe accumulate more cadmium compared to birds from the southern part of the continent. For instance, specimens from Sweden accumulated in their kidneys 2.95 mg/kg DW (median) (Frank 1986) and 2.1 mg/kg DW, and 5.19 mg/kg DW (medians) in the Netherlands (Hontelez et al. 1992; Jager et al. 1996). The results of our study, which showed cadmium levels at 1.67 mg/kg DW, are comparable to the values mentioned earlier. However, studies conducted in Southern Europe (Italy, Spain, and Portugal) have reported lower cadmium levels in the kidneys of Common Buzzards. For example, Battaglia et al. (2005) found a median level of 0.620 mg/kg DW, Naccari et al. (2009) reported a mean level of 0.55 mg/kg DW, Licata et al. (2010) found a median of 0.50 mg/kg DW, and Carneiro et al. (2014) reported a level of 0.865 mg/kg DW. The aforementioned geographical patterns in cadmium concentrations probably arise from differences in agriculture. In farms in northern and western Europe, much more phosphorus fertilizers are used, often contaminated with cadmium compared to the southern part of Europe (Alengebawy et al. 2021; Toth et al. 2016).

## Mercury

Mercury does not play any physiological function in organisms. The toxicity and transport of this element, and its incorporation into ecosystem pathways, depend on its physicochemical form (Scheuhammer et al. 2007; Kalisińska et al. 2019). Organometallic forms like methylmercury, phenylmercury, ethylmercury, and methoxyethyl mercury compounds are considered the most toxic forms (Ikingura and Akagi 1999; Scheuhammer et al. 2007; Jiskra et al. 2018). Results of many studies have indicated that avian kidneys do not play as significant a role in mercury accumulation as the liver. However, the role of kidneys in metabolizing methylmercury is emphasized (Ikingura and Akagi 1999; Scheuhammer et al. 2007; Dias dos Santos et al. 2021). Usually, higher mercury concentrations in the liver compared to the kidneys have been reported for most birds, with few exceptions (Horai et al. 2007; Kalisińska et al. 2019; Vizuete et al. 2019; Dias dos Santos et al. 2021). A study from Portugal has reported a lack of significant differences in mercury concentrations between the livers (1.39 mg/kg DW) and kidneys (2.09 mg/kg DW) of Common Buzzards (Carneiro et al. 2014). The previous study on liver mercury levels in wintering Common Buzzards in Eastern Poland (Kitowski et al. 2017b) has revealed that only adult females (2.31 mg/kg DW vs. 1.95 mg/kg DW), but not males (1.83 mg/kg DW vs. 2.76 mg/kg DW), accumulated more mercury in the livers compared to the kidneys.

Sex-specific differences in mercury concentrations in the internal organs of other species have been reported previously (Rajaei et al. 2010; Sepúlveda and Gonzalez-Acuna 2014; Misztal-Szkudlińska et al. 2018). Despite the sexual dimorphism in the Common Buzzard, no differences in mercury concentrations were observed in contrast to lead.

This study found no significant influence of age on the amount of mercury accumulated in the kidneys. Similarly, Castro et al. (2011) and Carneiro et al. (2014) reported no effect of age on kidney mercury levels in Common Buzzards and other raptors from the Iberian Peninsula. However, both studies showed that blood mercury concentrations were significantly higher in adults than in juveniles, which can be attributed to differences in diet and feather molting between these age groups (Carneiro et al. 2014).

Mercury tends to be highly bioavailable and mobile in wetland ecosystems serving as foraging areas for a variety of avian species including raptors. However, many studies have indicated weak bioavailability and mobility of mercury in terrestrial ecosystems (Hopkins et al. 2007; Kalisińska et al. 2019), which means that exploiting terrestrial prey does not promote mercury biomagnification in the kidneys. This helps to explain the relatively low accumulation of mercury in the kidneys of the studied Common Buzzards and in other terrestrial predatory birds (Kenntner et al. 2003; Houserova et al. 2005; Carneiro et al. 2014; Horai et al. 2007).

Almost complete elimination of mercury-based pesticides, massively used in agricultural practice in Poland in the past (Falandysz 1994; Kitowski et al. 2015) may also be associated with low mercury levels in the studied Common Buzzards.

## Arsenic

Arsenic is one of the most important environmental pollutants and a well-known carcinogen (Kaur et al. 2011). Fortunately, many studies have indicated that this metalloid does not have strong tendencies to accumulate in the key avian organs (Binkowski 2019; Vizuete et al. 2019). Concentrations of up to 0.25 mg/kg DW in bird kidneys are considered normal (Binkowski 2019). Our study confirms the arsenic inability to accumulate in key organs.

Only livers of 24.3% of studied individuals exceeded the aforementioned level, with a median value of 1.4 mg/kg DW. The maximum value recorded (2.62 mg/kg DW) was also far below the toxicity threshold of 41.6 mg/kg DW (Binkowski 2019). It is also worth noting the lack of correlation between As kidney concentrations and any other analyzed element (Fig. 3).

Other studies have also indicated a weak tendency for accumulation of arsenic in Common Buzzards with a maximum value of 0.588 mg/kg DW in Portugal (Carneiro et al. 2014) and 0.27 mg/kg DW in Sicily (Italy) (Naccari et al. 2009).

In this study no intergroup differences in the kidney concentrations of the considered metalloid were found. However, Naccari et al. (2009) have reported that Common Buzzard’s males accumulated significantly more arsenic compared to females (0.26 mg/kg DW vs. 0.19 mg/kg DW). Another study has reported that adult Common Buzzards showed significantly higher kidney concentrations of arsenic than juveniles (0.217 mg/kg DW vs 0.139 mg/kg DW), and females accumulated significantly more of this element than males (0.245 mg/kg DW vs 0.166 mg/kg DW) (Carneiro et al. 2014). The first phenomenon has been explained by the fact that over time there is local arsenic bioconcentration due to the chronic exposure of adult individuals to this element.

## Selenium

While kidneys may not have as large a capacity to accumulate selenium as livers do, selenium concentrations in the liver are 2–3 times higher and in kidneys ~ 1.8 times higher than in the consumed food (Albers et al. 1996). Nevertheless, kidneys are indicated to play an important role in accumulating this metalloid in vertebrate organisms (Pilarczyk et al. 2019).

The current study has shown significant positive correlations between concentrations of selenium and highly toxic elements such as mercury (*r*_s_ = 0.41) and cadmium (*r*_s_ = 0.51). This corresponds to the mitigating effect of selenium on vertebrate organisms exposed to the mentioned heavy metals reported by other authors (Jihen et al. 2008; Ohlendorf and Heinz 2009; Zwolak and Zaporowska 2012; Pilarczyk et al. 2019). It has also been observed that inorganic mercury and methylmercury can inhibit the selenoenzyme glutathione peroxidases (GSH-Px), which primary function is to protect the organism against oxidative stress (Branco et al. 2012; Pilarczyk et al. 2019). Other studies have shown that interactions between mercury and selenium in key avian organs are influenced by factors such as the chemical forms and concentrations of both elements in the ecosystems where the analyzed species live, the duration of exposure, and the dietary habits of these species (Ohlendorf and Heinz 2009; Pilarczyk et al. 2019). In some species, these interactions have been reflected by highly significant correlations between selenium and mercury levels in the kidneys (Hopkins et al. 2007). On the other hand, some researchers have not confirmed the relationship between the indicated elements (Wenzel and Gabrielsen 1995).

Despite the existence of numerous studies on the key organs of birds, there is no consensus among authors regarding the background selenium level in the avian kidney (Pilarczyk et al. 2019). Available data for poultry indicate that a range of 2.2–5.2 mg/kg DW may be considered background selenium kidney concentrations for birds (Puls 1994; Ohlendorf and Heinz 2009; Pilarczyk et al. 2019). On the other hand, the risk of selenium toxicity is present at concentrations in kidneys > 20–22 mg/kg (Ohlendorf and Heinz 2009; St Clair et al. 2015). Applying the above criteria, we found that none of the studied birds accumulated such high amounts of the considered metalloid in their kidneys (Table 1, Table 2). Their kidney selenium concentrations were also far from those (> 10 mg/kg DW) that could have induced at least sublethal effects (Ohlendorf and Heinz 2009). The above corresponds with data from other studies indicating selenium deficits in animal organisms, including birds, as well as deficits of this element in soils in the area of Poland, compared to other areas in Europe (Stec et al. 2005; Bombik et al. 2010; Pilarczyk et al. 2009, 2010; Chałabis-Mazurek and Wałkuska 2014).

## Sulfur

Sulfur is rarely measured in the viscera of vertebrates, with the liver being the primary focus. Vertebrates accumulate sulfur in the range of approximately 6–10,000 mg/kg DW in both the liver and kidneys (Kitowski et al. 2017a,b; Ribeiro et al. 2023). Nevertheless, White-tailed Eagles from the Baltic Sea have been found to have higher concentrations of this element in their livers—an average of 14,000 mg/kg DW (Falandysz et al. 2001). On the other hand, the significant role of sulfur in mitigating the impact of heavy metals on key organs of vertebrates is emphasized (Tamas and Martinoia 2006; Colovic et al. 2018). Our analyses revealed a significant correlation between lead and sulfur concentrations in the kidneys of Common Buzzards (Fig. 3). This reflects the role of sulfur in defense against heavy metal poisoning. It has been found that naturally occurring amino acids with sulfur and peptide glutathione residues serve as important agents of organism defense against heavy metal impact (Tamas and Martinoia 2006). Heavy metal-induced oxidative stress may be reduced, and the antioxidant capacities in heavy metal-exposed animals can be altered by sulfur-containing compounds (Tamas and Martinoia 2006; Caylak et al.^2007^; Colovic et al. 2018). Sulfur-containing amino acids (SAA) play a crucial role in the cell’s antioxidant system. Importantly, some of them are also metabolized in the kidneys (Scammahorn et al. 2021). In addition to their valuable antioxidant action, SAA can act as chelating agents for heavy metals, aiding in the removal of toxic metals and offering beneficial effects (Tamas and Martinoia 2006; Colovic et al. 2018). This explains the observed increase in sulfur levels with the increase in lead concentrations in the studied kidneys. This, of course, does not exclude sulfur interacting with other heavy metals such as cadmium, mercury, and zinc. Especially since, for the latter element, we also demonstrated a significant positive correlation with sulfur levels in the studied kidneys.

## Zinc

Zinc concentrations in avian kidneys range from 83 to 133 mg/kg DW, with levels of 300–800 mg/kg DW considered toxic (Kosik-Bogacka and Łanocha-Arendarczyk 2019). However, even higher levels of this metal in the kidneys (960 mg/kg DW) have been reported for free-ranging wild birds, resulting from environmental zinc poisoning (Sileo et al. 2003). Concentrations below 83 mg/kg DW indicate deficiencies of this element in the avian organism (Kosik-Bogacka and Łanocha-Arendarczyk 2019). Our analyses showed that 21 out of 41 birds (51.2%) had kidney concentrations indicative of zinc deficiencies, which may reflect issues with prey availability for half of the studied population. We attribute the above to the decline in densities of main prey (voles) during the autumn–winter period. A study on Common Buzzards from Sicily, collected between April and December, reported that out of 11 Eurasian Buzzards analyzed, only 2 birds (18.2%) had kidney zinc levels indicating deficiencies of this element (Licata et al. 2010). Conversely, Yipel et al. (2023) found no individuals with kidney concentrations exceeding 83 mg/kg dry weight in injured raptors from southern Turkey, collected throughout different times of the year. For Golden Eagles (*Aquila chrysaetos*), the mean was 19.0 mg/kg wet weight (25.3 mg/kg dry weight), and for Sparrowhawks (*Accipiter nisus*), the mean was 18.1 mg/kg wet weight (24.1 mg/kg dry weight).

A positive correlation between Zn and Pb concentrations in the kidneys of Common Buzzards has been found in this study. Such a relationship was also demonstrated in the livers of owls from South Korea (Kim et al. 2008). This reflects the importance of zinc in mitigating the impact of lead in vertebrate organisms because lead absorption is highly influenced by zinc levels. Consequently, the negative effects of lead on vertebrate key organs may be alleviated by adequate zinc concentrations (Wani et al. 2019, 2021). It has been demonstrated that dietary zinc supplementation prevents histological dysfunction of the kidneys and regulates parameters related to oxidative stress. Overall, zinc mitigates the toxic influence of lead on kidney tissue as an antioxidant and nephroprotective factor (Soussi et al. 2018). However, as our research on wild birds indicates, the availability of food resources may play a significant role in this regard.

## Copper

In contrast to the liver, there is limited data regarding kidney concentrations of copper in birds (Łanocha-Arendarczyk and Kosik-Bogacka 2019). Birds do not accumulate as much copper in their kidneys as in their livers. At least in some water-associated groups, the amounts of accumulated copper in livers can reach > 1000 mg/kg DW (Schummer et al. 2011; Komosa et al. 2012; Łanocha-Arendarczyk and Kosik-Bogacka 2019). In the present study, we did not observe sex differences in the accumulation of this metal in the kidneys. Such differences are also extremely rarely found in other species (Stewart et al. 1994; Aazami and KianiMehr 2018).

It has been found that terrestrial raptors, including Common Buzzards, generally do not accumulate large amounts of copper in their kidneys. Higher levels of copper are found only in the kidneys of wetland herbivores (such as swans) and piscivorous birds (such as herons) (Łanocha-Arendarczyk and Kosik-Bogacka 2019).

However, even a concentration level of 50 mg/kg DW in the kidney may cause a nephrotoxic effect in birds (Frank and Borg 1979; Horai et al. 2007; Łanocha-Arendarczyk and Kosik-Bogacka 2019). In the studied Common Buzzards, we did not detect kidney concentrations causing nephrotoxic effects (Table 1, Table 2). Additionally, very often similar and liver concentrations of copper are noted in the same species (Stewart et al. 1994; Horai et al. 2007; Lucia et al. 2010; Milaimi et al. 2016). Less frequently, other patterns have been reported, for example, for Mute Swans (*Cygnus olor*), concentrations several times higher than kidney concentrations were noted (Frank and Borg 1979).

The average copper concentrations found in our study (12.17 mg/kg DW) strongly correspond with results of other studies on diurnal and nocturnal raptors reporting kidney concentrations ranging from 13.1 to 14.5 mg/kg DW (Jager et al. 1996; Horai et al. 2007; Kim and Oh 2012).

## Strontium

Soft tissues play a marginal role in the accumulation of Sr, as evidenced by our results for Common Buzzard kidneys and analyses of kidneys and livers of other avian species (Nam et al. 2005; Skoric et al. 2012; Ansara-Ross et al. 2013; Rodríguez-Álvarez et al. 2022). Significantly higher levels of this metal have been found in avian samples such as eggshells, feathers, and bones (Mora et al. 2007; Jakubas et al. 2019; Skoric et al. 2012; Rodríguez-Álvarez et al. 2022).

Strontium has a chemical similarity to calcium, which enables the replacement of calcium by strontium in biomineralization processes (Blaschko et al. 2013). Intestinal absorption and kidney filtration studies revealed that vertebrates process strontium in much the same way as calcium (Samachson et al. 1966; Vezzoli et al. 1998; Blaschko et al. 2013). On the other hand, similar to lead and cadmium, this metal is nephrotoxic (Staessen et al. 1992; Zhang et al. 2023). It competes with calcium to bind to calcium-binding receptors (Handlogten et al. 2000). Disturbances in the calcium balance might therefore result in the formation of kidney stones, with strontium playing a significant role in their initiation process. Conversely, environmental lead exposure is a risk factor for nephrolithiasis (Blaschko et al. 2013; Singh and Rai 2014; Hara et al. 2016). These results help to explain the elevated strontium levels we observed in the kidneys of adult females, that may be exposed to higher levels of lead contamination (more frequent foraging on carrion of animals shot with lead ammunition compared to smaller males), often resulting in disruption of normal kidney function, which may also result in the accumulation of higher amounts of strontium.

## Manganese

Previous analyses on a large sample of various raptors species have indicated that mean/median kidney manganese levels are most often < 6 mg/kg DW (Kalisińska and Budis 2019). Higher concentrations have been less frequently encountered (Horai et al. 2007; Kalisińska et al. 2008; Mahmood et al. 2022). These findings are in concordance with our results (Table 1, Table 2). Kidney manganese concentrations in birds appear to be smaller than their liver values (Horai et al. 2007; Kalisińska and Budis 2019; Mahmood et al. 2022).

Positive correlations between manganese and lead concentrations, but negative correlations between manganese and cadmium have been found in the kidneys of the studied birds (Fig. 3). The same pattern has been found for lead and manganese, but the opposite pattern has been shown for owls from South Korea (Kim et al. 2008). In the livers of Grey Heron (*Ardea cinerea*) and Schrenck’s Bittern (*Ixobrychus eurhythmus*) from South Korea, increasing cadmium concentrations have been accompanied by increasing manganese concentrations (Kim and Oh 2013). These results confirm the ability of manganese to reduce cellular uptake of cadmium or to promote cellular resistance to cadmium exposure (Martin et al. 2006). A positive correlation between manganese and iron concentrations in the kidneys was observed in the studied Buzzards. This may be associated with the positive relationship between blood manganese levels and hemoglobin, as well as the important role of the kidneys in the metabolism and accumulation of both elements (Kim et al. 2017; Chen et al. 2018; Glogowski et al. 2021; Liu et al. 2021). Additionally, several similarities were found in transport of these two elements in vertebrates (Liu et al. 2021). The studied Common Buzzards accumulated an average of 8.57 mg/kg manganese in their kidneys. Other species have accumulated comparable amounts of manganese ranging from 4.02 to 10.44 mg/kg DW (Horai et al. 2007; Kalisińska et al. 2008). In this context, somewhat unexpected are the data from Pakistan (Punjab province) for the Black Kite (*Milvus migrans*) showing only 1.53 mg/kg DW in the kidneys (Mustafa et al. 2015), while data for the same species from the same province indicated 10.2 mg/kg DW manganese in the kidneys accompanied by 4.7 mg/kg DW in feathers, 11.8 mg/kg DW in livers, and 15.9 mg/kg DW in muscles (Mahmood et al. 2022). Perhaps the first of these studies has involved sick individuals kept in captivity because they also had surprisingly low levels of manganese in feathers, livers, and muscles: 0.63 mg/kg DW, 2.19 mg/kg DW, and 2.33 mg/kg DW, respectively.

### Iron

It has been found that avian kidneys are organs inferior to livers in terms of iron accumulation (Falandysz et al. 1988, 2001; Jager et al. 1996; Kalisińska et al. 2009; Kosik-Bogacka et al. 2019). A quite wide range of the considered metal accumulation in the kidneys of diurnal raptors, ranging from ~ 200 to ~ 1200 mg/kg DW have been reported (Falandysz et al. 1988, 2001; Jager et al. 1996, Kalisińska et al. 2006, 2008, 2009). The results of our analyses regarding the accumulated iron content in the kidneys (median: 753.2 mg/kg DW) fully correspond to the results within this range. In contrast to the liver (Kalisińska et al. 2008; Kitowski et al. 2017b), cases of hyperaccumulation (> 5000 mg/kg DW) are extremely rare in the kidneys of raptors, including Common Buzzards (Jager et al. 1996). In other species, such as herons, noticeably lower amounts of kidney iron, with concentrations not exceeding 40 mg/kg DW have been reported (Mansouri et al. 2012). It should also be noted that typically the amount of accumulated iron in raptor kidneys, as in the liver, is greater than zinc and copper (Kalisińska et al. 2009; Kosik-Bogacka et al. 2019), which we also confirmed in our research.

The levels of iron and other elements in avian kidneys demonstrate their mutual relationships and interactions (Fox et al. 1984; Groten et al. 1991; Kalisińska et al. 2009; Kosik-Bogacka et al. 2019). In our research, we observed that low iron concentrations in the kidneys are accompanied by high kidney concentrations of cadmium and nickel. The negative effects of cadmium on levels of kidney iron have been reported for growing chicks of Common Pheasants *Phasianus colchicus* (Świergosz and Kowalska 2000).

Therefore, in situations of increased cadmium uptake by avian kidneys, it is essential to ensure proper iron supply to vertebrate organisms (Fox et al. 1984; Groten et al. 1991). This was also observed in the kidneys of Common Buzzards studied by Kalisińska et al. (2009), as well as in our study (Fig. 3), where a positive correlation between iron and copper concentrations was noted.

## Vanadium

Fertilizers, fossil fuels, municipal sewage sludge, or vanadium products from mine tailings serve as the main sources of vanadium associated with anthropogenic activity. The military sector activity is a significant anthropogenic source of vanadium in the environment (Sladkova et al. 2015; Wnuk 2023).

Vanadium is an extremely rarely analyzed element in the organs of birds. Kidneys of vertebrates are the organ with the highest vanadium accumulation in the body, followed by spleen, liver, and bones, muscles (Hansen et al. 1982; Agusa et al. 2005). Various levels of vanadium accumulation have been reported for avian kidneys. Relatively high levels have been reported for ducks, for example, in the kidneys of ducks such as Spotbill Duck (*Anas poecilorhyncha*), Mallards (*Anas platyrhynchos*), Pintail (*Anas acuta*), and Wigeon (*Anas penelope*) from East Japan, an average of: 3.69–8.11 mg/kg DW vanadium, while only 0.39–3.69 mg/kg DW of this element was found in their livers (Mochizuki et al. 1999). In this study, we also found: 1.66 mg/kg DW vanadium in kidneys, whereas in the previous study on the livers of Common Buzzards from E Poland, only 0.13–0.18 mg/kg DW has been found (Kitowski et al. 2017b). Other researchers have reported lower amounts of vanadium in the kidneys of the same species compared to those shown above. For example, Agusa et al. (2005) report only 0.11 mg/kg DW and 0.063 mg/kg DW, respectively, for the kidneys of adult and young individuals.

Our study has shown that females accumulated less vanadium in their kidneys compared to males. We believe that similar to lead, the greater vanadium concentration in the kidneys of females is related to the phenomenon that Common Buzzard females more frequently outcompete other scavengers interested in carrion of game animals not recovered by hunters. Vanadium is commonly used in alloys to resist rusting, abrasion, and high temperatures, in the manufacture of armaments, including gun barrels to increase service life and performance (Pavel et al. 2020; Ścibior et al. 2021; Koniorczyk and Zieliński 2023). Thus, vanadium from gun barrels enters through shot ammunition to the bodies of hunted animals and then to foraging on them Common Buzzards. It has been found that the use of various types of alloys made with the use of vanadium for the production of various types of weapons, including firearms, also causes noticeable soil contamination with vanadium in areas where this type of weapon is intensively exploited (Sladkova et al. 2015).

## Limitation of our study

Our study has some limitations. The most important one is that we used a relatively small number of non-randomly collected kidneys in our analyses. Most of the samples analyzed originated from individuals in poor condition, close to death, and delivered to the veterinary clinic. However, it should be noted that the samples analyzed in this study were collected from a relatively large area and were diverse in age and sex. Further studies based on a larger number of kidney samples are needed.

## Conclusions

In this study, significant sex differences in the accumulation of some elements in kidneys of Common Buzzards wintering in East Poland were found. Females accumulated more lead and vanadium than males. These differences may be explained by inter-sex size dimorphism affecting the diet composition of both sexes. Larger females may more frequently forage on carrion of shot animals contaminated with lead and vanadium. Our results indicate that accumulation patterns of chemical compounds in living organisms can be very complex and category-specific. Thus, sampling of different groups (age, sex, etc.) with their specific characteristics (hormonal, physiological, ecological, etc.) is crucial for a better understanding of the exposure of organisms to various elements and compounds.

## Data Availability

The data will be available on a reasonable request.
